# Perforation of inferior vena cava and duodenum by strut of inferior vena cava filter: A case report

**DOI:** 10.1097/MD.0000000000017835

**Published:** 2019-11-22

**Authors:** Hyun Oh Park, Jun Young Choi, In Seok Jang, Jong Duk Kim, Jong Woo Kim, Joung Hun Byun, Sung Hwan Kim, Jun Ho Yang, Seong Ho Moon, Ki Nyun Kim, Dong Hun Kang, Jae Jun Jung, See Min Choi, Ji Yoon Kim, Chung Eun Lee

**Affiliations:** a Department of Thoracic and Cardiovascular Surgery, Gyeongsang National University Hospital, Gyeongsang National University College of Medicine, Jinju; b Department of Thoracic and Cardiovascular Surgery, Gyeongsang National University Changwon Hospital, Gyeongsang National University College of Medicine, Changwon; c Department of Urology; d Department of Anesthesiology and Pain Medicine, Gyeongsang National University Hospital, Gyeongsang National University College of Medicine, Jinju, Republic of Korea.

**Keywords:** complications, pulmonary embolism, vena cava filters, venous thrombosis

## Abstract

**Introduction:**

An Inferior vena cava (IVC) filter is an intravascular filter that is implanted into the IVC to prevent pulmonary embolism in medical, surgical, and trauma patients. The insertion of an IVC filter is a relatively safe procedure, but rarely may be associated with symptomatic perforation of the IVC wall, particularly in the long term.

**Patient concerns and diagnosis:**

A 74-year-old-woman with a medical history of IVC filter insertion visited the emergency department complaining of abdominal pain. A computed tomography scan showed perforation of the IVC wall and penetration into the duodenum by one of the filter's struts.

**Interventions:**

We performed a laparotomy to remove the IVC filter.

**Outcomes:**

Postoperatively, the patient was admitted to the general ward. On hospital day 12, she was discharged without any complications. We followed her up and computed tomography did not show any abnormal findings six months after discharge.

**Lessons:**

There is currently no evidence testifying to the benefits of IVC filter removal. Detailed, evidence-based guidelines on the indications, timing and procedure for IVC filter removal are needed. Documenting cases of long-term complications of IVC filter s such as in this patient serve to accelerate the publication of updated guidelines and are aimed at improving outcomes of similar cases in the future.

## 1 Introduction

Pulmonary embolism (PE) is a blockage of one of the pulmonary arteries by a blood clot or foreign material (embolus) that has traveled through the bloodstream from elsewhere in the body to the lungs. The most common cause of PE is deep vein thrombosis (DVT) in the lower extremities. PE is a serious, potentially life-threatening complication of DVT.^[1]^ Anticoagulation is the treatment of choice for venous thromboembolism, but in patients who have contraindications to the use of anticoagulants, are not compliant, or suffer from recurrent venous thromboembolism, an IVC filter may decrease morbidity and mortality by reducing the incidence of PE.^[2]^ Current IVC filter types can be classified as permanent or temporary/retrievable filters. With the advent of the latter type, the use of IVC filters has increased significantly.^[1,2]^ The most common complications of IVC filters are tilt, migration, fracture, deviation, and DVT.^[1]^ A perforation of the IVC wall is a rare, but serious complication. We report a case of IVC and duodenum perforation by a filter strut.

## 2 Case report

A 74-year-old woman presented to our emergency department with abdominal and back pain that had started two days earlier. The patient further complained of nausea and vomiting and described a worsening of her abdominal pain with postural changes. The physical examination of the patient showed diffuse abdominal tenderness, but no rebound tenderness. Her blood pressure was 190/90 mm Hg and the heart rate 78 beats per minute without the use of inotropic drugs. The laboratory findings were as follows: hemoglobin 12.9 g/dL, white blood cell count 8.07 × 10^9^/L, and C-reactive protein, 0.4 mg/L. The patient's medical history included deep vein thrombosis and pulmonary embolism with subsequent placement of Celect IVC filter (Cook Medical, Bloomington, Indiana, USA) approximately eight years ago. The patient had undergone IVC filter implantation in another hospital, and the exact reason for the insertion could not be ascertained.

**Figure 1:**
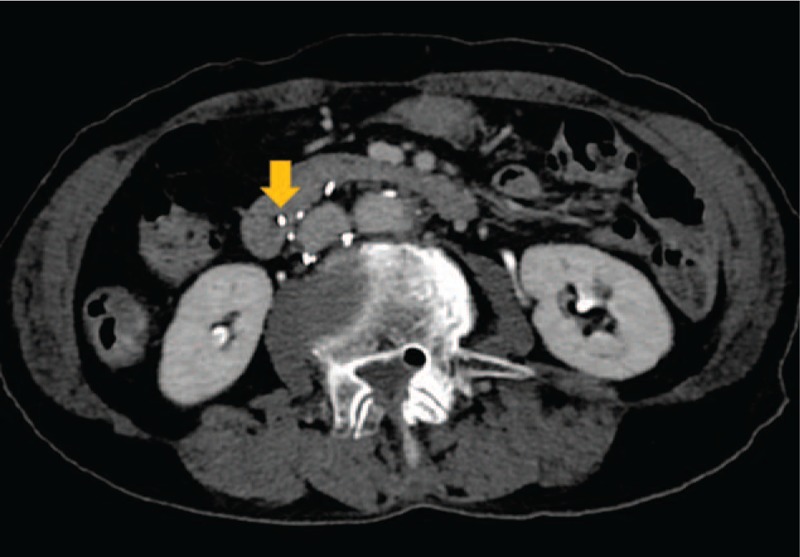
A 74-year-old-woman with abdominal and back pain, nausea and vomiting. Computed tomography shows one of the struts (arrow) of the inferior vena cava (IVC) filter has migrated through the IVC wall into the duodenum.

Computed tomography (CT) revealed a perforation of the IVC wall by one of the filter's struts and penetration into the duodenum. We did not identify another cause of abdominal pain in our examination (Fig. 1).

**Figure 2:**
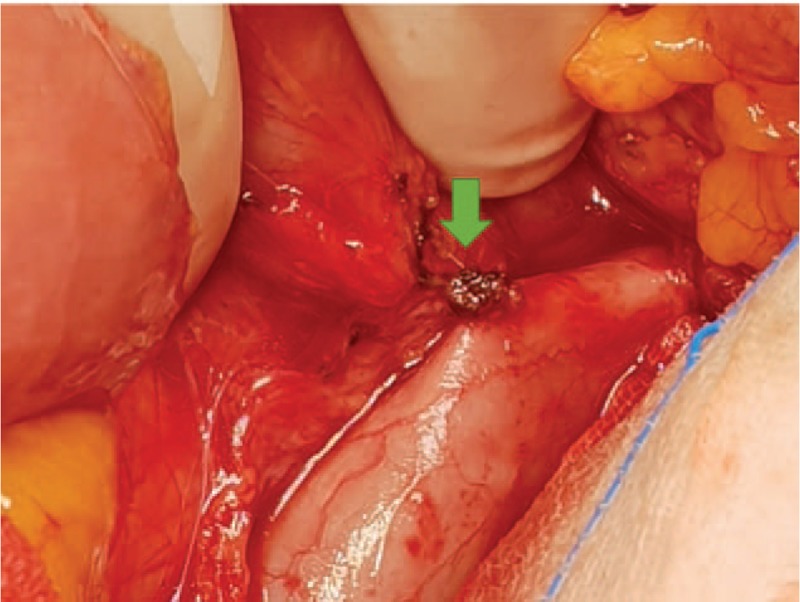
Intraoperative findings: The arrow indicates where the filter strut protrudes out of the inferior vena cava and penetrates into the duodenum.

**Figure 3:**
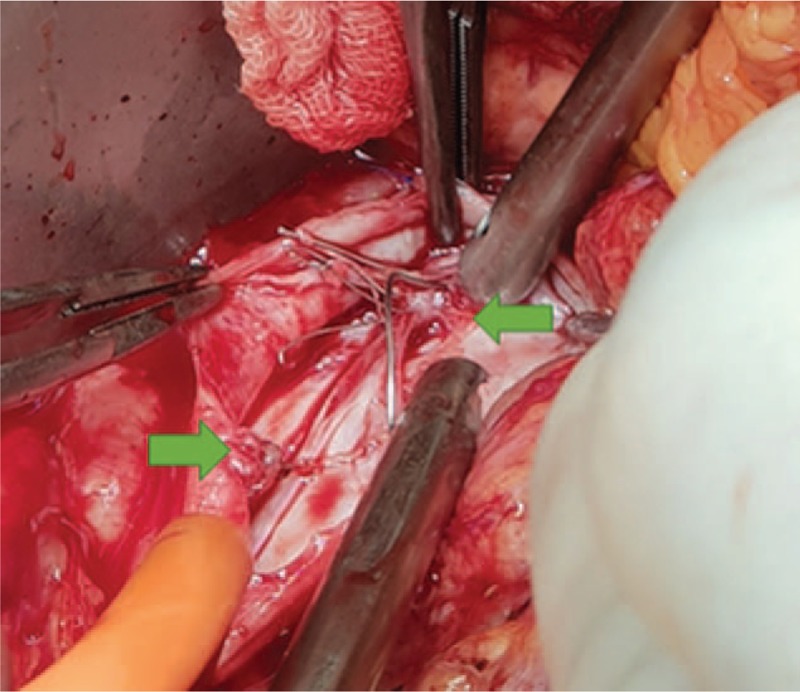
Intraoperative findings: View of the inferior vena cava showing intimal fibrosis (arrows).

Our first attempt to remove the IVC filter through cavography failed. The patient consecutively underwent median laparotomy. On retraction of the duodenum to the right, one strut of the filter could be seen protruding the vessel wall and penetrating into the duodenum (Fig. 2). The strut was removed from the duodenum entirely, and the perforation site was sutured primarily with Vicryl (Ethicon Inc., Somerville, NJ). The IVC was dissected at the level of the left renal vein and the confluence of common iliac veins to permit safe clamping. An IVC clamp was then used to clamp the IVC proximally just below the renal vein. A second IVC clamp was placed above the iliac vein confluence. We then opened the IVC and found that the IVC filter was obscured by intimal fibrosis. The IVC filter struts were separated with wire cutters and removed without significant IVC injury (Fig. 3). The IVC was repaired with a running 5-0 Prolene (Ethicon Inc., Somerville, NJ) suture, and the abdomen was closed in standard fashion. The patient's postoperative hospital stay was uneventful without any complications. On hospital day 12, she was discharged. We followed her up for six months. We followed the patient up and her CT did not show any abnormal findings six months after discharge.

## 3 Discussion

An IVC filter is a vascular device that is inserted into the IVC to prevent the traveling of blot clots from a DVT to the lungs and cause possibly life-threatening PE.^[1]^ The first IVC filter, the Mobin-Uddin umbrella, was introduced in 1967 and had to be placed via a thoracotomy.^[3]^ In 1984, a Greenfield filter for percutaneous insertion was developed, and later retrievable IVC filters were introduced.^[4]^ In 2003, the Food and Drug Administration (FDA) approved amendments to three so far permanent filters to allow their retrievable versions to be used, and, since then, an increasing number of new types and versions of IVC filters have been developed. Since the use of IVC filters in general has grown exponentially in recent years, retrievable filters are expanding at similar rates.^[5]^

There have been many studies on IVC filters, but for a long time, no clear indications for their insertion or removal had been established. In 2006, the Society of Interventional Radiology recommended that IVC filters should be inserted in the following groups of patients at the highest risk of PE: 1) Proven venous thromboembolism (VTE) in patients who have contraindications for or suffered complications of anticoagulation; 2) Patients with recurrent VTE despite adequate anticoagulation therapy.^[5]^

With the increasing use of IVC filters, reports of complications are also increasing. Typical complications of IVC filter insertion include filter tilt, displacement, migration, fracture and/or embolization and thrombosis.^[1]^ Occasionally, caval penetration may also occur. Filter penetration is defined as extension of filter components more than 3 mm outside the vena caval wall.^[6]^ Penetration of the vena cava has been reported to cause injury to adjacent organs such as the aorta, duodenum, and large intestine.^[6,7]^ The overall incidence of vena caval wall penetration has not been reported.^[8]^ Our patient received a Celect retrievable IVC filter. Charles et al.^[9]^ reviewed the records of 115 patients and found that 57 filters (49.6%) were successfully removed, and two cases (1.74%) of penetration occurred. Some patients with penetration are asymptomatic and may have symptoms such as abdominal discomfort, abdominal pain, fever, melena, and hematochezia.^[6,7]^ The diagnosis of caval penetration is complex, and examination via cavography or CT is recommended.^[5]^ Treatment of vena caval wall penetration caused by an IVC filter depends on the presence or absence of symptoms. Symptomatic penetration should be treated by radiologic intervention or surgery.^[8]^ The Society of Interventional Radiology recommends prevention rather than treatment of complications caused by IVC filters.^[5]^ Most adverse complications are considered to occur when IVC filters remain in the body for a long term. Hence, the Society of Interventional Radiology encourages all physicians responsible for the treatment of patients with retrievable IVC filters to consider removing the filter immediately once PE protection is no longer needed.^[5]^

In conclusion, the evidence of the benefits of IVC filter removal is still limited at this point, and additional, more detailed guidelines for IVC filter removal are required. Documenting cases of long-term complications of IVC filters as in this patient serve to accelerate the publication of updated guidelines and help to improve the outcomes of similar cases in the future.

## Acknowledgments

We acknowledge the outstanding contributions of the technicians and nursing staff at the Gyeongsang National University Hospital, Korea.

## Author contributions

**Conceptualization:** Hyun Oh Park, Jun Young Choi, Chung Eun Lee.

**Data curation:** Hyun Oh Park, In Seok Jang, Jong Duk Kim, Jong Woo Kim, Joung Hun Byun, Sung Hwan Kim, Jun Ho Yang, Seong Ho Moon, Ki Nyun Kim, Dong Hun Kang.

**Project administration:** Chung Eun Lee.

**Resources:** Joung Hun Byun, Sung Hwan Kim, Dong Hun Kang, Jae Jun Jung, See Min Choi, Ji Yoon Kim, Chung Eun Lee.

**Supervision:** Jun Young Choi, Jong Woo Kim, Chung Eun Lee.

**Validation:** Ji Yoon Kim.

**Visualization:** Jae Jun Jung, See Min Choi.

**Writing – original draft:** Hyun Oh Park, Chung Eun Lee.

**Writing – review & editing:** Hyun Oh Park, Chung Eun Lee.
